# Do technically efficient surgeons continue to provide active clinical services in a university hospital?

**DOI:** 10.1371/journal.pone.0254515

**Published:** 2021-07-09

**Authors:** Yoshinori Nakata, Yuichi Watanabe, Hiroshi Otake, Akihiko Ozaki

**Affiliations:** 1 Teikyo University Graduate School of Public Health, Tokyo, Japan; 2 Waseda University Graduate School of Economics, Tokyo, Japan; 3 Department of Anesthesia, Showa University School of Medicine, Tokyo, Japan; 4 Department of Surgery, Joban Hospital, Fukushima, Japan; Yale University, UNITED STATES

## Abstract

It is difficult for university hospitals to recruit and retain technically efficient surgeons because their missions include teaching and research as well as clinical services. The authors hypothesized that technically efficient surgeons do not continue to provide active clinical services in a university hospital. The authors collected data from all the surgical procedures performed at Teikyo University Hospital from April 1 through September 30 in 2013–2018. The dependent variable was defined as a length of each surgeon’s active clinical services measured by month. Data envelopment analysis was employed to calculate each surgeon’s technical efficiency score. Five control variables were selected; experience, medical school, surgical volume, gender, and academic ranks. Multiple regression analysis was performed. Efficiency scores had significantly negative association with length of active clinical services. Experience and surgical volume had significantly positive association with length of active clinical services. The other coefficients of control variables were insignificant. Technically efficient surgeons provide shorter active clinical services in a university hospital.

## Introduction

Operating room efficiency is an important concern in most hospitals, including university hospitals and academic medical centers. It is considered to depend on surgeons’ technical efficiency because they usually utilize the longest time portion of the operating room time. Recruiting and retaining technically efficient surgeons are the keys for the hospitals to survive in the increasingly fierce healthcare market competition. However, it may be difficult for university hospitals and academic medical centers to recruit and retain technically efficient surgeons because their missions include not only clinical services but also teaching and research.

We previously reported that technically efficient surgeons have shorter tenures at a university hospital [1]. According to our literature search on Medline, this has been the first and only research that focused on the relationship between surgeons’ length of active clinical services and their technical efficiency. However, our report only demonstrated the negative correlation between efficiency scores and length of employment measured by the year. The length of employment measured by the year was either 1, 2, 3, 4, or 5, and the data granularity of the dependent variable was too coarse to be conclusive [1]. In order to evaluate technically efficient surgeons’ active clinical services more accurately, we have decided to measure their length of services by the month in this study. The present study is an extension of our previous one.

We again hypothesized that technically efficient surgeons do not continue to provide active clinical services in a university hospital. The purpose of this study is to more precisely determine the relationship between surgeons’ length of active clinical services and their technical efficiency by using multiple regression analysis on actual surgical data.

## Materials and methods

The Teikyo University Institutional Review Board approved our study. Anonymity of the data was strictly maintained by de-identification by the research team. No consent was obtained because our present study was a retrospective observational study and the need for consent was waived by the Institutional Review Board. We used similar methods of data collection, analysis framework and statistical analysis to our previous study except for the following two points [1]. First, we added the newest data from the year of 2018 for our analysis. Second, we measured surgeons’ length of active clinical services by the month in this study.

### Data

Teikyo University Hospital is located in metropolitan Tokyo, Japan, serving a population of ~1,000,000. It has 1,152 beds and has a surgical volume of approximately 9,000 cases annually. It has thirteen surgical specialty departments. We collected data from surgical records in the Teikyo University Hospital electronic medical record system. We collected data from all the surgical procedures performed in the main operating rooms of Teikyo University Hospital from April 1 through September 30 in 2013–2018. Because of our time and budget constraints, we collected data only for six months (April-September) each year. We extracted the necessary information from the Teikyo University Hospital electronic medical record system [2, 3].

Exclusion criteria for surgery were as follows. First, surgical procedures performed under local anesthesia by surgeons were excluded. Second, oral and dermatologic surgical procedures were excluded because most of those cases were minor surgeries that are clinically different from major surgeries. Third, the surgical procedures were excluded if the patients die within one month after surgery to maintain a constant quality outcome of surgery. Although death is not a major determinant of quality in all surgical patients, it was the only available quality indicator that was common to all surgical procedures. Fourth, the surgical procedures that were not reimbursed under the surgical payment system were excluded because it was impossible to compute efficiency scores without the data on surgical fees (see below). Fifth, the surgical procedures were excluded if their records were incomplete for any reason although this is a disadvantage of retrospective analysis [2, 3].

### Efficiency scores (Independent variable)

The method to calculate surgeons’ technical efficiency was similar to that described in our previous studies [2, 3]. Data envelopment analysis (DEA) is a measure of technical efficiency that takes into account multiple inputs and multiple outputs. DEA has been widely used to measure technical efficiency of various health care entities [4, 5]. Individual surgeons’ technical efficiency can also be measured by efficiency scores calculated from DEA [6]. We defined surgeons’ technical efficiency as follows: technically efficient surgeons maximize their output while minimizing their input utilization.

Technical efficiency is an economic term which is in contrast to allocative efficiency. A decision making unit (DMU) is defined as the entity that is responsible for converting inputs into outputs in DEA [7]. Technical efficiency reflects the ability of a DMU to produce the maximum amount of output given a set of inputs while allocative efficiency reflects the ability of a DMU to use inputs in optimal proportions given their respective prices [8]. Generally speaking, senior surgeons are responsible for the technical efficiency while healthcare managers are responsible for the allocative efficiency in healthcare settings.

#### Microeconomic model in technical efficiency calculation

We employed output-oriented Charnes-Cooper-Rhodes model of DEA under the constant returns-to-scale assumptions, which does not require an a priori specification of a function [9]. In this analysis we focused on the surgeons’ activity and their clinical decision. We defined in this study the DMU as a surgeon with the highest academic rank that scrubbed in the surgery (a senior surgeon). All the inputs and outputs are under the control of a DMU. Inputs were defined as (1) the number of medical doctors who assisted surgery, and (2) the time of surgical operation from skin incision to skin closure (surgical time). The output was defined as the surgical fee for each surgery. Japan has maintained a universal health insurance system, and most health care providers are reimbursed on a fee-for-service basis according to the fee schedule that set prices uniformly at the national level. The same fee schedule is enforced for all plans and all the surgeons studied [10]. It is classified as K000- K915 in the Japanese surgical fee schedule, and is called “K codes.” Each surgical procedure is assigned to one of the K codes which correspond with surgical fees. The fee is identical regardless of who (a senior surgeon or a surgical trainee) performs surgery as long as they have medical licensure, how many assistants they use, or how long it takes to complete surgery. The additional reimbursements for expensive surgical devices, such as auto suture devices, were excluded. Other fees for blood transfusion, medications, special insurance medical materials and anesthesia were also excluded because they were not under the control of senior surgeons.

We added all the inputs and outputs of the surgical procedures for each DMU in each year during the 6-month study period and calculated her /his efficiency scores using DEA-Solver-Pro Software (Saitech, Inc., Tokyo, Japan). The efficiency scores all lie between 0 and 1, and the most efficient surgeons are given the score of 1. All the surgeons in the sample are given an efficiency score in each year. We used as independent variables the mean efficiency scores of the years when surgeons performed surgery as a senior surgeon at Teikyo University Hospital.

### Dependent variable

The dependent variable was defined as a length of active clinical services of each surgeon. It was measured as the number of months when a surgeon performed at least one surgery as a senior surgeon at Teikyo University Hospital. We analyzed the data for 36 months from 2013 through 2018. Therefore, the dependent variable is one of the integers from 1 to 36.

### Control variables

We selected five control variables that are available to us and may influence surgeons’ length of active clinical services. We previously demonstrated that they were not significantly correlated with surgeons’ efficiency scores [6].

#### Experience and medical school

Most surgeons analyzed in this study publish their medical schools that they attended and their years of graduation in directories and/ or websites. Surgeons’ experience was defined as the number of years since medical school graduation on the last date of surgical procedure at Teikyo University Hospital.

Seven former imperial universities are highly regarded among medical schools in Japan. They attract the brightest candidates, and their entrance examination is notoriously competitive every year. Surgeons who graduated from former imperial universities are expected to be more academically oriented than those who did not. We classified medical schools into two categories by whether or not they are former imperial universities. If they are former imperial universities, we assigned a dummy variable of 1; otherwise, we assigned a dummy variable of 0.

#### Average surgical volume per six months

Average surgical volume was defined as the mean of surgical cases that a surgeon performed during the six-month study period (April-September) in each year. This information was extracted from the Teikyo University Hospital electronic medical record system.

#### Gender and academic ranks

Teikyo University Hospital makes surgeons’ gender and academic rank publicly available in its website for patients’ convenience. We assigned a dummy variable of gender, with female = 1 and male = 0.

Academic ranks at the last year when a surgeon performed surgery were recorded from the Teikyo University Hospital website. We assigned two dummy variables of academic ranks. The first dummy variable (Rank (Professor)) was assigned with full professor = 1 and otherwise = 0. The second dummy variable (Rank (Associate Professor)) was assigned with associate professor = 1 and otherwise = 0.

### Statistical analysis

We used Stata Data Analysis and Statistical Software (Stata 14, StataCorp LP, College Station, Texas, U.S.A.) for our statistical analysis. We performed multiple regression analysis using an ordinary least squares model for our data. The dependent variable was length of active clinical services measured by the month. The independent variables were mean efficiency scores and five control variables. A p-value < 0.05 was considered statistically significant.

## Results and discussion

### Results

We analyzed a total of 17,227 surgical cases performed by 313 surgeons in 36-months of study from 2013 through 2018. Efficiency scores were calculated for all the surgeons in each year, and the mean of the efficiency scores for each surgeon was calculated.

The characteristics of the dependent and independent variables are shown in Table 1. The mean length of active clinical services was 11.8 months out of the 36-month study period. The mean efficiency score was 0.29 in all the 313 surgeons studied. We could obtain information on medical schools and experience only from 247 and 227 surgeons, respectively; other surgeons did not make these data publicly available. The reason for this lack of publicity was unknown to us, and we could not tell how it confounded our results.

**Table 1:** Characteristics of dependent and independent variables.

**Length of Active Clinical Services (months) (n = 313)**		11.8 ± 10.8	(1–36)
**Mean Efficiency Scores (n = 313)**		0.29 ± 0.22	(0.03–1.00)
**Experience (years) (n = 227)**		18.6 ± 9.6	(2–44)
**Medical School (n = 247)**	Imperial Universities	46	(18%)
	Other	201	(82%)
**Surgical Volume (cases/ 6 months) (n = 313)**		14.7 ± 17.0	(1.0–99.8)
**Gender (n = 313)**	Female/ Male	39/ 274	(13%/ 87%)
**Academic Rank (n = 313)**	Full Professors	40	(13%)
	Associate Professors	32	(10%)
	Other	241	(77%)

Data are presented as mean ± SD (range) or absolute values (%).

The results of ordinary least squares model multiple regression analysis were shown in Table 2. We performed multiple regression analysis for 222 surgeons who published information on both medical schools and experience. Efficiency scores had significantly negative association with length of active clinical services (p = 0.006). If a surgeon’s efficiency score increases by 0.1, her/ his length of active clinical services will decrease on average by 0.7 months. Experience and surgical volume had positive association with length of active clinical services (p = 0.001 and p = 0.000, respectively). If a surgeon’s experience increases by one year or if a surgeon’s average surgical volume increases by one case per six months, her/ his length of active clinical services will increase on average by 0.3 months. The coefficients of medical schools, gender, and academic ranks were statistically insignificant (p > 0.05).

**Table 2:** Results of multiple regression analysis using an ordinary least squares model.

Dependent variable: Length of active clinical services (months)			
	Coefficients	95% Confidence Interval	p-value
**Efficiency Score** *	- 7.336 ± 2.651	- 12.56, - 2.11	0.006
**Experience** *	0.321 ± 0.094	0.136, 0.506	0.001
**Medical School**	- 0.136 ± 1.732	- 3.552, 3.279	0.937
**Surgical Volume** *	0.329 ± 0.045	0.240, 0.417	0.000
**Gender**	0.527 ± 1.560	- 2.547, 3.602	0.735
**Rank (Professor)**	- 1.729 ± 2.482	- 6.620, 3.163	0.487
**Rank (Associate Professor)**	4.216 ± 2.260	- 0.238, 8.670	0.063

Data are presented as mean ± robust standard error.

* indicates that the coefficient is significantly different from zero (p < 0.05).

## Discussion

From our ordinary least squares model multiple regression analysis, we demonstrated that technically efficient surgeons have shorter length of active clinical services at a university hospital. The longer their experiences were and the larger their surgical volumes were, the longer they provide active clinical services in a university hospital. Their medical schools, gender, or academic ranks did not have any significant predictive values for their length of active clinical services. This finding demonstrated that technically efficient surgeons do not continue to provide active clinical services in a university hospital after controlling their experience, surgical volumes, academic ranks, medical schools and gender.

The present study more clearly confirmed the findings of our previous one demonstrating a university hospital cannot retain technically efficient surgeons because the sample size of the present study was larger and its data granularity was finer than in our previous one. As a result, its p-value (p = 0.006) was smaller than that of the previous one (p = 0.011) [1]. There has been no other study that evaluates the relationship between surgeons’ length of active clinical services and their technical efficiency using DEA.

There are several possible reasons for our findings. First, university hospitals usually aim to achieve not only clinical services but also academic and educational excellence. Technically efficient surgeons who focus only on clinical excellence may not fit the missions of university hospitals and may leave them early. Second, university hospitals serve as educational institutes. It is possible that surgeons work in university hospitals until they become technically efficient. Once they become technically efficient enough, they may seek employment in private practice, which may provide better monetary rewards. Third, technically efficient surgeons may be promoted to leadership positions, which reduces their time for active clinical services in a university hospital. They may spend more time at their administrative offices than in the operating rooms. Fourth, proficiency in teaching and research does not necessarily correspond with clinical excellence. Surgeons who meet the academic and educational criteria of university hospitals may not be as technically efficient as surgeons outside university hospitals.

Surgeons with long experience continued to provide active clinical services in our university hospital. This may be because surgeons with long experience are older and likely to have an established academic career. In addition, they may have few employment opportunities outside a university hospital. This might have led to their longer active clinical services at a university hospital. The small surgical volume led to the shorter length of active clinical services. Surgeons who are less active in surgery have few surgical opportunities at a university hospital. It is natural that the small surgical volume is associated with shorter active clinical services.

### Limitations

There are some limitations in our study. First, this is a study conducted in a single large teaching hospital in Tokyo, Japan. Our hospital and surgeons may not represent all the surgeons. However, there is an advantage to studying surgeons’ technical efficiency in a single hospital. Since one of the significant resource inputs is ancillary services such as operating room nursing practices and availability of support personnel, all these factors are held constant in a single hospital. Comparing surgeons in different hospitals can be misleading if some ancillary services are more efficient than others. By comparing surgeons in the same institution, they all face the same systemic advantages and disadvantages of ancillary services [11].

Second, we only evaluated surgeons’ technical efficiency in the operating rooms, not their academic and educational achievements. Academic ranks may serve as a proxy variable for these achievements although they did not have significant effects on the length of active clinical services. It would be difficult to identify the exact causes of our findings without this information. However, the exact data on their academic and educational achievements were unavailable to us.

Third, we simply considered the number of assistants without taking their experience into account. It is obvious that a full surgeon of lesser rank is not equivalent to junior surgical trainees in assisting surgery. An unequal distribution of residents with different experience is also likely. On services like cardiovascular, thoracic and neurosurgery, more senior trainees need to be present, and it is unlikely that a junior trainee would get to do anything technically, or perhaps even scrub. However, the detailed data were unavailable [12].

Fourth, we used postoperative death within a month as the only quality indicator of clinical outcomes, and excluded the surgical procedures if the patients die within one month after surgery. We did not include any other patient factors in our analysis. It is true that the quality of the outcome of surgery is not constant just because the patient did not die within one month after surgery. However, our sample included different kinds of surgical procedures. Death was the only available outcome measure that is common to all different kinds of surgery although we understand that it is imperfect [3].

Fifth, generalization of our results may be limited because this study was conducted in a university hospital in Japan. Different countries practice different human resource management under different healthcare systems. Our results may not be applicable to another part of the globe. However, the present study is the only research except our previous one that evaluated the relationship between surgeons’ length of active clinical services and their technical efficiency using DEA. The future research direction will be to replicate our study in another country with a different healthcare system.

Sixth, our definition of technical efficiency has limitations. Surgical assistants are usually surgical trainees in a university hospital. If a senior surgeon is very eager in education and lets a number of trainees assist her/ his surgery, she/ he will suffer in technical efficiency in our definition. Our definition of technical efficiency may not reflect surgeons’ duty in a university hospital.

Seventh, we only demonstrated the association between surgeons’ length of active clinical services and technical efficiency. The causality was impossible to establish from our ordinary least squares model multiple regression analysis. In order to clearly compare the length of active clinical services, we need to disaggregate the data and compare among different stratified groups of surgeons regarding efficiency scores, experience and surgical volume. However, this disaggregation will make our sample size too small to draw any statistically meaningful conclusions because we had only 222 surgeons in total.

Eighth, the choice of control variables largely depended on the data availability rather than their actual influence on the length of active clinical services in a university hospital. Especially, gender and medical school may be considered unrelated to the dependent variable. However, since there are general concerns about the data protection, it was impossible to collect more meaningful personal data on surgeons from open data sources.

Ninth, we did not include as an independent variable the hospital revenue that surgeons made although it is easier to calculate than surgeons’ technical efficiency. We previously found that hospital revenue may serve as a proxy variable for surgeons’ technical efficiency [13]. However, we focused on calculating surgeons’ technical efficiency directly, and did not use any proxy variable because of its multicollinearity among independent variables in the multiple regression model. It might be possible that surgeons who make more hospital revenues have shorter tenues in university hospitals. Further study will be required to demonstrate the relationship between hospital revenue and active clinical services.

## Conclusions

We again demonstrated that technically efficient surgeons provide shorter active clinical services in a university hospital. Although this was a single-center retrospective study in Japan, it was the only available research except our previous one that evaluated the relationship between surgeons’ length of active clinical services and their technical efficiency using DEA.
